# Impact of Electronic Health Record Use on Cognitive Load and Burnout Among Clinicians: Narrative Review

**DOI:** 10.2196/55499

**Published:** 2024-04-12

**Authors:** Elham Asgari, Japsimar Kaur, Gani Nuredini, Jasmine Balloch, Andrew M Taylor, Neil Sebire, Robert Robinson, Catherine Peters, Shankar Sridharan, Dominic Pimenta

**Affiliations:** 1 Guy's and St Thomas' NHS Trust London United Kingdom; 2 Tortus AI London United Kingdom; 3 Manchester University NHS Foundation Trust Manchester United Kingdom; 4 Barts Health NHS Trust London United Kingdom; 5 Great Ormond Street Hospital London United Kingdom

**Keywords:** electronic health record, cognitive load, burnout, technology, clinician

## Abstract

The cognitive load theory suggests that completing a task relies on the interplay between sensory input, working memory, and long-term memory. Cognitive overload occurs when the working memory’s limited capacity is exceeded due to excessive information processing. In health care, clinicians face increasing cognitive load as the complexity of patient care has risen, leading to potential burnout. Electronic health records (EHRs) have become a common feature in modern health care, offering improved access to data and the ability to provide better patient care. They have been added to the electronic ecosystem alongside emails and other resources, such as guidelines and literature searches. Concerns have arisen in recent years that despite many benefits, the use of EHRs may lead to cognitive overload, which can impact the performance and well-being of clinicians. We aimed to review the impact of EHR use on cognitive load and how it correlates with physician burnout. Additionally, we wanted to identify potential strategies recommended in the literature that could be implemented to decrease the cognitive burden associated with the use of EHRs, with the goal of reducing clinician burnout. Using a comprehensive literature review on the topic, we have explored the link between EHR use, cognitive load, and burnout among health care professionals. We have also noted key factors that can help reduce EHR-related cognitive load, which may help reduce clinician burnout. The research findings suggest that inadequate efforts to present large amounts of clinical data to users in a manner that allows the user to control the cognitive burden in the EHR and the complexity of the user interfaces, thus adding more “work” to tasks, can lead to cognitive overload and burnout; this calls for strategies to mitigate these effects. Several factors, such as the presentation of information in the EHR, the specialty, the health care setting, and the time spent completing documentation and navigating systems, can contribute to this excess cognitive load and result in burnout. Potential strategies to mitigate this might include improving user interfaces, streamlining information, and reducing documentation burden requirements for clinicians. New technologies may facilitate these strategies. The review highlights the importance of addressing cognitive overload as one of the unintended consequences of EHR adoption and potential strategies for mitigation, identifying gaps in the current literature that require further exploration.

## Introduction

**Figure 1:**
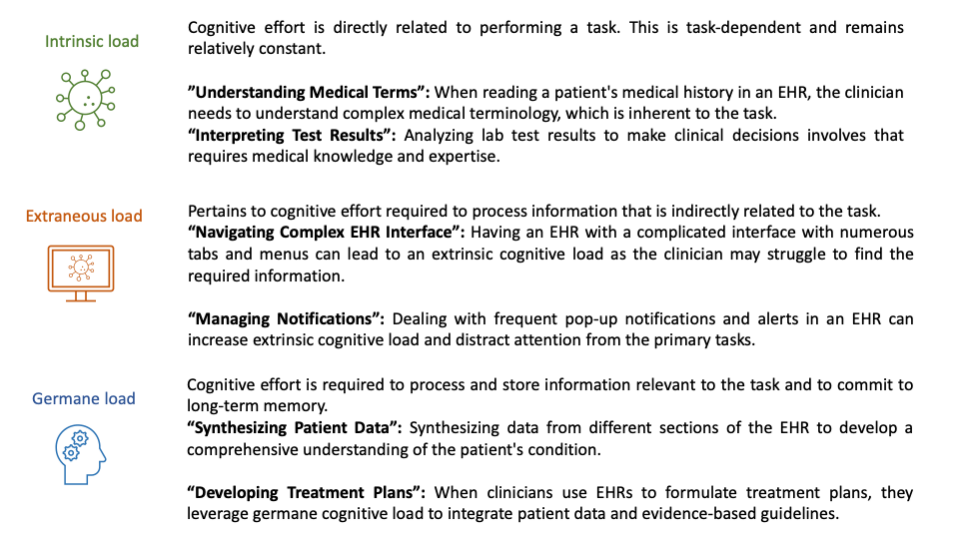
Factors contributing to cognitive load and examples of how they are affected when clinicians use EHRs. EHR: electronic health record.

Sweller [1] defined cognitive load as “the amount of mental effort required to process and store information in working memory.” The cognitive load theory argues that completing a task requires a complex interplay between sensory inputs, working memory, and long-term memory [1]. The working memory helps interpret the sensory input and then commits processed information into long-term memory. This psychological theory stipulates that while both sensory and long-term memories can handle large volumes of input data, working memory has a comparatively limited capacity and can keep 3 to 5 items in mind at a time [2]. When the amount of information exceeds this given capacity, it leads to cognitive overload. For any given task, there are 3 main factors that contribute to its perceived cognitive load: intrinsic, extraneous, and germane [1]. Figure 1 provides a description of each cognitive load with examples of how they are affected when clinicians use electronic health records (EHRs).

Physician burnout refers to a state of chronic physical and emotional exhaustion experienced by clinicians, often due to prolonged stress and heavy workloads. It is a growing concern in the medical sector, both in the United Kingdom and globally, and it affects individual health care professionals as well as the health care system as a whole. Key aspects include emotional exhaustion, demoralization, and a reduced sense of accomplishment [3].

Contributing factors to burnout include unremitting workloads, administrative burdens, emotional stress, work-life imbalance, the lack of autonomy, and insufficient support systems. Physicians who work long hours and have high patient loads can experience physical and mental fatigue. Additionally, administrative tasks, paperwork, and EHR requirements add to the workload, causing frustration [4].

According to a report from the World Health Organization, the average life expectancy globally has risen by 6 years, from 66.8 years in 2000 to 73.4 years in 2019 [5]. It is estimated that 1 in 3 adults have multiple long-term conditions [6]. With advancements in science and technology, there are now more diagnostic and treatment options available, which require additional monitoring and follow-up. Consequently, physicians face an increased cognitive load due to the larger volume of information they need to process from complex patients to deliver high-quality care.

Simultaneously, the extraneous load experienced by clinicians is influenced by the presentation of this information. When information is disorganized, unnecessary, or incomplete, clinicians are exposed to a higher extraneous load, placing a greater demand on their working memory [7], which in turn has a knock-on effect on the germane load. This effect is most pronounced among early-career clinicians with a significant amount of new material to learn [7].

Working memory is also attenuated in the presence of physiological or emotional stress [8], which in recent times has become increasingly more common among clinicians following the COVID-19 pandemic, leading to widespread burnout.

Although EHR systems are widely used in health care settings worldwide, there are not enough comprehensive studies evaluating their advantages and disadvantages or examining how they can be improved. In a systematic review, Moy and colleagues [9] aimed to identify the studies on physician and nursing burnout related to using EHR systems. They found that 40% of the 35 studies meeting their inclusion criteria had mentioned clinical burnout. However, they noted a lack of standardized and validated measures to assess the documentation burden related to EHR use. There is also a lack of objective measures to assess the cognitive load associated with using EHRs.

In summary, the increasing cognitive load experienced by physicians is a sum of the increasing complexity of the information presented, the quality and clarity of that presentation, as well as the emotional and psychological context in which the information is received [1]. The usability of EHRs, including their design, interoperability, and various regulatory requirements, impacts this cognitive burden. By working constantly above the cognitive load threshold, clinicians may exhibit symptoms of cognitive overload, which is considered an immediate precursor to burnout [10]. This narrative review examines the existing literature on cognitive overload experienced by health care professionals, specifically in relation to their use of EHRs and the associated burnout. The review also explores potential solutions that could help reduce the EHR-related cognitive load and improve the well-being of clinicians.

## Use of EHRs in Health Care

The digitization of health care has been a growing trend over the past decades, which has seen most patient data transferred from paper records to EHRs. The use of EHR dates back to the 1960s but was only limited to government use [11]. Since the 1970s, EHR systems have been developed with hierarchical or relational databases for various indications, such as to help with hospital billing and scheduling systems, to help improve medical care, and for use in medical research. As computers have become more accessible, larger health care organizations have begun using them to gather patient information [12]. This shift has gradually expanded to encompass nonclinical tasks such as administration, medicolegal work, research, and education [13], which have also increased in demand and complexity over time.

Poor physician acceptance and the lack of incentives, in addition to high costs and errors associated with data entry, hindered EHR implementation uptake in the 1990s, and thus, digital records were not widespread [14]. By the 2000s, countries such as the United States and the United Kingdom began implementing national projects to digitize paper records [12]. The UK government attempted to create the National Programme for Information Technology [15] in 2002 to create a universal EHR system for the entire United Kingdom. The nationwide initiative was centered around 3 main objectives: lifelong EHRs, 24/7 web-based access by public health care professionals, and seamless information sharing throughout all sectors of the National Health Service [16]. The project failed to meet its objectives, and digitization became fragmented and slow once again [17]. However, by 2022, a total of 86% of the UK hospital trusts had successfully transitioned from paper notes to a digital system (although only a minority have enterprise-wide EHR capability), with the figure expected to reach 90% by December 2023 [18]. In the United States, the *Health Information Technology for Economic and Clinical Health Act* was signed into law in February 2009 to promote the adoption of and meaningful use of health information technology (HIT) as part of the American Recovery and Reinvestment Act. Financial incentives that were allocated as part of the *Health Information Technology for Economic and Clinical Health Act* led to a significant increase in the adoption of EHRs in the United States [14].

## Factors Impacting the Usability of EHR Systems

The usability of EHR systems continues to be a major concern, whereby clinicians are subjected to too much or too little information, preprogrammed workflows, and multiple alerts [19]. There have been problems with chaotic, nonintuitive visual displays and numerous default settings that might not be relevant for a given task or patient [20]. Navigating through the same information includes unnecessary steps, for example, multiple clicks and duplicated information [21]. Users experience higher fatigue, leading to potential room for errors and decreased efficiency [22,23]. In addition to documentation and chart review, managing inbox tasks has been noted as one of the significant burdens for clinicians [24]. Receiving excessive notification has been shown to cause alert fatigue, leading to missing important information and poor patient outcomes [25]. Although clinical decision support systems have been introduced to enhance patient care, excessive use of interruptive clinical decision support systems in the EHRs can lead to alert fatigue and reduced effectiveness. Chaparro and colleagues [26] have described how interruptive alerts can increase cognitive burden and lead to reduced acceptance of the alert and an increase in the number of errors.

Several factors have been highlighted as contributing to the use of EHR and physician well-being. Nguyen and colleagues [27] studied this in a systematic review, where they found that EHR-related physician well-being is determined by multiple factors, including EHR usability, EHR system features, and physician-level characteristics and beliefs.

The sheer volume of data that a physician can access during a specific clinical encounter proves challenging [28]. As an example, Hill and colleagues [29] found that emergency health care physicians see an average of 2.4 patients per hour and use 4000 mouse clicks in a 10-hour shift. This can result from a combination of poor EHR design and information overload and adds to physician stress [30,31]. Information overload is a part of the 5 main hazards of “information chaos” alongside information underload, information scatter, information conflict, and erroneous information [32]. Clinicians are then required to spend more effort to filter through the information, clarify conflicting documentation, or reassess potentially erroneous information, leading to excess workload and adverse outcomes on not only patient care and health systems but, more importantly, clinician well-being [33].

Gal and colleagues [34] studied this in a pediatric intensive care unit where they calculated that each patient generated an average of 1460 new data points in a 24-hour period. Pediatric intensive care unit attending physicians cared for an average of 11 patients during the day and 22 patients overnight, resulting in exposure to 16,060 (range 11,680-18,980) and 32,120 (range 23,360-37,960) individual data points during the day and night, respectively.

Wanderer and colleagues [35] have described how optimal data visualization in various specialties can lead to improved decision-making for clinicians and more efficient use of their time. Many EHR vendors use visual analytic systems to improve physician workflow and reduce medical errors [36].

Blink rate, measured using eye-tracking technology, has been associated with cognitive workload. Visual tasks that require more focused attention and working memory load have been shown to reduce blink rate [37]. A decreased blink rate has been found to occur in EHR-based tasks that require more cognitive workload [38].

The NASA Task-Load Index (NASA-TLX) is a widely used questionnaire to assess perceived workload (available in Multimedia Appendix 1) [39]. It consists of 6 questions, which can be rated from 1 to 10. Nurses rated their perceived workload from 0 (very low) to 10 (very high).

Using blink rate in addition to the NASA-TLX, Mazur and colleagues [40] tested the implications of the EHR usability interface in a study where they assigned tasks, including the review of medical test results for 20 and 18 individuals using baseline and enhanced EHR versions, respectively, that provided policy-based decision support instructions for next steps. Interestingly, they found that the baseline group had poorer performance and higher cognitive load compared with those who used the enhanced version, suggesting the importance of improving the usability of EHRs to address issues such as clinician burnout and patient safety events.

Harry and colleagues [7] studied the direct relationship between cognitive load with physician burnout in a national sample of US physicians. Using the NASA-TLX, they had responses from 4517 (85.6%) of the 5276 physicians included in the survey. The median age of the physicians was 53 years; 61.8% were male, 37.9% were female, and 0.3% were other gender; and 24 specialties were identified. They identified a dose-response relationship between physician task load and the risk of burnout independent of age, gender, practice setting, and hours worked per week.

To demonstrate a more accurate association between EHR use and stress, Yen and colleagues [41] used blood pulse wave monitoring (previously used as a surrogate for chronic stress) in addition to NASA-TLX on 7 nurses during 132 hours of work. They found that the nursing staff spent 45.54 minutes using EHR during a 4-hour shift, which was much more than the time spent on any other communication or hands-on activities. In addition, the nurses’ stress when using EHR was associated with higher perceived physical demand and frustration.

The level of EHR-related burnout has also been shown to be in part influenced by physician specialty. In a large study that used assessing questionnaires among physicians in various specialties with over 25,000 respondents, the investigators found the level of burnout ranged from 22% to 34% by specialty [42]. The specialties with the highest levels of burnout were family medicine (34%) and hematology or oncology (33%). The specialties with the lowest levels of burnout were psychiatry (22%) and anesthesiology (24%). After adjusting for confounding variables, physicians with 5 or fewer hours of weekly out-of-hours charting were twice as likely to report lower levels of burnout than those with 6 or more hours. Those who agree that their organization has performed well with EHR implementation, training, and support were also twice as likely to report lower levels of burnout than those who disagreed. This highlights the importance of training and support following the implementation of EHR for their optimal use.

In a scoping review, Muhiyaddin and colleagues [43] studied the causes and consequences of physician burnout related to the use of EHRs. Reviewing 30 eligible studies out of 500, they identified 6 main causes that are related to physician burnout, including EHR documentation and related tasks, poor design of EHR systems, workload leading to overtime work, inbox alerts, and alert fatigue. Not surprisingly, physician burnout was associated with a low quality of care, behavioral issues, and mental health complications, as well as career dissatisfaction and a reduction in patient safety and satisfaction.

In a survey of 640 clinicians from 3 institutions, with 282 (44.1%) responses to 105 questions, Kroth and colleagues [30] identified 7 EHR design and use factors associated with high stress and burnout. These were information overload, slow system response times, excessive data entry, inability to navigate the system quickly, note bloat, interference with the patient-clinician relationship, fear of missing something, and notes geared toward billing.

Another study [44] aiming to quantify burnout due to the use of HIT used a survey sent to 4197 physicians, where 1792 responded (response rate: 42.7%). They found that HIT-related stress was measurable, prevalent, and specialty related. About 70% of physicians with EHRs experienced HIT-related stress in their sample, and the presence of any of the 3 HIT-related stress measures independently predicted burnout symptoms among respondents. In particular, those with time pressures for documentation or those doing excessive “work after work” on their EHR at home had approximately twice the odds of burnout compared to physicians without these challenges. Time spent after hours on the EHR and the volume of inbox messages have been found to relate to physician exhaustion [45].

Using live observational design and NASA-TLX surveys, Khairat and colleagues [46] assessed the effect of EHRs on emergency department attending and resident physicians’ perceived workload, satisfaction, and productivity through completing 6 EHR patient scenarios. They found that EHR frustration levels are significantly higher among more senior attending physicians compared with more junior resident physicians. Among the factors causing high EHR frustrations are (1) remembering menu and button names and commands use; (2) performing tasks that are not straightforward; (3) system speed; and (4) system reliability.

## Advantages and Disadvantages of Using EHRs

### Overview

As highlighted in the previous section, despite their potential benefits, there have been growing concerns that EHRs also have detrimental effects. Here, we summarize some of the advantages and disadvantages of using EHRs.

Information overload is a significant concern when using EHRs [47-49]. Various studies also suggest a correlation between the usability of the EHR and cognitive load and burnout among clinicians [34,50-52]. Clinicians feel that work-life balance, satisfaction rates, attrition, and burnout are all affected due to the continuous daily interaction with EHR systems [22,53-55].

### Advantages

The transition from paper-based medical records to EHRs has been perceived as a positive development in several areas [9,56]. In addition to being easily accessible, EHR systems have been shown to improve communication between clinicians and enhance the continuity of care [57,58]. They can lead to better-informed decisions due to the availability of data and avoid the duplication of diagnostic testing [59]. However, a review of the impact of EHR use on enhancing medication safety, one of the biggest risks to patient care, has shown only modest improvements [60].

EHRs also contain a high volume of clinical data, providing us with multiple opportunities to conduct research and audit [13,59]. A good example of this in the United Kingdom is OpenSAFELY, a secure, transparent, and open-source software platform for the analysis of EHR data [61]. During the COVID-19 pandemic, scientists and statisticians could use the data available on this platform to provide insights into population demographics most at risk of death following COVID-19 infection, which aided with the national policy strategy for prioritizing care [62-65].

### Disadvantages

Over the past decade, there has been a reported increase in burnout levels among clinicians, with one potential factor being the introduction of EHR systems [23,34]. The introduction of EHRs has resulted in changes in workflow, with frontline clinicians taking on administrative tasks such as ordering tests, correcting notes, and placing referrals. This has led to increased cognitive load, which is often overlooked [66,67].

On a day-to-day basis, clinicians face time constraints; administrative load; and consequently, elongated workdays. The current documentation methods used in EHRs are under scrutiny by clinicians due to the perceived poor quality of user interfaces, ultimately leading to burnout [52]. Factors such as increased structured documentation requirements, physician order entry, inbox management, and patient portals contribute to more work that is not direct face time with patients [19,68].

Inflated documentation also extends to the excessive use of templates and copy-and-paste workflows in EHR systems that introduce data that are neither required nor accurate [48,49,69]. EHRs allow information to be copied from almost anywhere in the record to another section. This can save time and allow clinicians to focus on clinical tasks rather than documentation; however, it comes with its own challenges. Erroneous information can be copied and pasted without editing, leading to data integrity issues and diagnostic errors [70]. This also creates room for false assumptions and inferred incorrect information between different health care professions, perpetuating previous inaccuracies. It might also sanction junior clinicians to rely solely on readily available information rather than conducting a thorough history and examination for themselves and constructing their own differential thought processes [71].

Although thorough documentation is key for clinical care, there has been a rise in complex and lengthy documentation of content that is required for billing purposes, quality improvement measures, avoiding malpractice, and signs of compliance [72]. In countries such as the United States, the regulatory requirements for data entry beyond what is required for direct patient care can contribute to an increasing workload [73]. Examples of these include collecting data for claim submission, prior authorization, billing, and quality reporting. In addition, a lack of interoperability between EHR systems can result in clinicians not having access to adequate patient information and fragmented care [74]. Often the clinical needs to spend a significant amount of time to obtain this information from various medical records between different health care organizations and sometimes even within one facility. Textbox 1 summarizes some of the advantages and disadvantages of using EHRs.

**Advantages and disadvantages of using electronic health records (EHRs).**

**Advantages of using EHRs**

- Improved communication between clinicians
- Remote access to clinical records enhances care delivery
- Convenient access to patient information for clinicians
- Facilitates research and audit through a high volume of clinical data storage

**Disadvantages of using EHRs**

- Information overload leading to cognitive overload
- Increased cognitive load due to EHRs can contribute to feelings of exhaustion and burnout
- Continuous interaction with EHRs affects work-life balance and may lead to burnout
- Complex and lengthy documentation required for billing and quality reporting can be cumbersome
- Poor quality of user interfaces in EHRs leads to clinician burnout
- Excessive use of templates and copy-and-paste workflows can lead to data integrity issues
- The lack of interoperability between EHR systems can lead to missed information and duplication of investigations

## Overcoming EHR-Related Burnout

Health care organizations and policy makers worldwide are increasingly recognizing the importance of addressing clinician burnout [75]. Here, we have summarized some of the interventions recommended in the literature that can reduce various types of cognitive load and potentially clinician burnout related to EHR use.

Dymek and colleagues [24] have made a case for producing an evidence base to reduce EHR-related clinician burden. Describing documentation, chart review, and inbox tasks as some of the key contributing factors causing burnout, they have made suggestions to help overcome these challenges. Some of these approaches include using speech recognition software and natural language processing to help with documentation and the generation of progress notes; the use of natural language processing and machine learning to process, filter, and rank patient information so that the attention can be paid to where it is most needed; and the use of better inbox design by involving clinicians in their development. Understanding the workflow of the clinicians and involving them in the design of the EHR have been shown to positively impact its usability and user satisfaction [76].

Several studies have reviewed alert burden in EHRs and described potential solutions on how to manage them effectively. One of the very interesting and useful recommended suggestions is developing an Interruptive Alert Stewardship by implementing metrics to assess the alert burden and their effectiveness in improving outcomes [26]. McGreevey and colleagues [77] have comprehensively described reasons for alert fatigue and suggest that organizations develop an alert governance specific to their needs. They propose that key stakeholders including clinicians, informatics, information technology, and administration groups need to participate in developing the alert governance and oversee the design and purpose of alerts. They also recommend using a checklist to assess the purpose and justification of alerts and suggest using metrics to assess their effectiveness and efficiency. Organizations such as Geisinger Health System and Penn Medicine have successfully improved their EHR alert to help with clinician well-being [77].

Clinicians have specific and feasible suggestions for reducing EHR-related burdens, such as providing high-quality EHR training; having an on-site EHR support team; involving support staff or scribes in the documentation process; and, importantly, obtaining physician input and feedback in improving EHRs [27]. Future efforts should focus on implementing the strategies and upgrades requested by these frontline users.

In a recent systematic review, Kang and Sarkar [78] looked at interventions that have been used to reduce EHR-related burnout. The study identified 3 primary interventions, including the use of scribes, EHR training, EHR modifications, and a combination of training and modifications. The use of scribes has been overall well received by clinicians and patients and, in some cases, led to increased productivity, but there were downsides, in particular, the cost, which would be difficult to overcome in smaller centers. EHR training had varying outcomes, with some studies showing a reduction in documentation time, whereas others did not demonstrate this benefit. Nevertheless, subjective EHR proficiency increased, which could help improve clinicians’ perception of EHR.

The study [78] has also examined several EHR modification techniques, such as data entry automation technology, improving EHR workflows, reducing unnecessary inbox alerts, and providing support teams to resolve EHR issues promptly. These interventions resulted in positive outcomes, such as a reduction in documentation time ranging from 18.5 to 60%, improved documentation quality and completion rate, decrease in data errors, and subjective EHR usability and satisfaction. However, these positive effects did not lead to a significant reduction in physician burnout. The authors suggest that this could be due to the fact that although EHR enhancements can improve some aspects of the clinician’s workflow, they probably do not address the defects in the EHR usability, which contribute to burnout. In addition, there are other factors contributing to burnout, such as overall workload, organizational culture, and work-life balance that extend beyond EHR systems.

Improving interoperability and health information exchange through understanding the barriers, appropriate incentives, and legislation can facilitate the clinicians’ workflow, reduce workload, and enhance patient safety [79].

In 2020, The Office of the National Coordinator for Health Information Technology published a report outlining strategies to reduce regulatory and administrative burden related to the use of HIT and EHR systems [80]. The report focuses on the challenges of EHR and HIT-related burden, which hinder the achievement of interoperability. They recognize that these burdens increase the time and expense clinicians must invest in interacting with EHRs, reducing the value of information, and diverting resources from patient care. They propose a framework for trusted exchange among health information networks to reduce clinician burden while benefiting patients and the health care system.

The National Academy of Medicine published a potential roadmap for EHR optimization and clinician well-being [81]. They have described several strategies currently available that can improve the usability of EHRs, such as EHR optimization, in-basket management techniques, documentation strategies, team-based workflow, and EHR training, as well as the use of artificial intelligence and add-on applications that can help with interoperability, automation, and decision support tools in the future. Gandhi and colleagues [82] have described how the use of artificial intelligence can reduce the cognitive workload by helping with data gathering, documentation, and decision support. They also suggest useful methods to assess the impact of these technologies.

## Gaps in the Current Literature

Given the increasingly interconnected digital ecosystem and the complexity of health care systems, which are influenced by physical, emotional, and human factors, it can be challenging to attribute specific outcomes to any particular technology.

Therefore, to maximize the benefits of new tools, it is essential to create frameworks for scientifically assessing the impact of any technology used.

By using user-friendly interfaces, customization options, and context-sensitive information presentation, EHRs can streamline data management [83]. Incorporating decision support tools, data visualization techniques, and smart documentation practices further enhances health care staff’s ability to focus on patient care, reducing the risk of errors and burnout [36]. EHR optimization to support clinical workflow and real-life working is a key to uplifting the well-being of health care professionals.

Addressing physician burnout requires systemic changes, including improving work environments with a renewed focus on teamwork, reducing administrative burdens, providing support, and promoting work-life balance within health care organizations.

Individual strategies, such as self-care, stress management, and professional pastoral help, are crucial for clinicians to mitigate and recover from burnout [84]. This will support the well-being of the health care workforce and ensure ongoing high-quality care delivery for all.

As our patients and work environments become more complex and more health technology products become available, it is crucial that we assess their impact through studies and engaging with our health care staff and patients throughout all stages of their development and use [85].

## Conclusion

The use of EHR systems may provide benefit for centralizing patient data and simplifying the process of reviewing records, requesting laboratory and imaging tests, and reviewing results, as well as conducting clinical audits, research, and quality improvement projects.

However, there is a noticeable difference in the quality of various EHR systems health care organizations use. Many of these EHR systems do not communicate with each other, keeping data isolated in silos. Our review highlights the cognitive load that their use places on clinical staff, which is not always considered. Improving the design, user interface, and data visualization or retrieval of EHR systems can help to reduce cognitive load, support working memory, and potentially reduce physician workload while enhancing patient care.

## Appendix

Multimedia Appendix 1 NASA Task-Load Index questionnaire.

## Abbreviations

EHR: electronic health record
HIT: health information technology
NASA-TLX: NASA Task-Load Index
