# Supplementary material for: Impact of Electronic Health Record Use on Cognitive Load and Burnout Among Clinicians: Narrative Review
Source: JMIR Med Inform. 2024 Apr 12;12:e55499. doi: 10.2196/55499 (PMC11053390; doi:10.2196/55499)
Supplement: Multimedia Appendix 1 [file medinform_v12i1e55499_app1.docx]

NASA Task-Load Index questionnaire

| **Item** | **Endpoints** | **Description** |
| --- | --- | --- |
| Mental demand | 1-10  Low/High | How much mental and perceptual activity was required (e.g., thinking, deciding, calculating, remembering, looking, searching, etc.)? Was the task easy or demanding, simple or complex, exacting or forgiving? |
| Physical demand | 1-10  Low/High | How much physical activity was required (e.g., pushing, pulling, turning, controlling activating etc was the task easy or demanding slow or brisk slack or strenuous, restful or laborious? |
| Temporal demand | 1-10  Low/High | How much time pressure did you feel due to the rate or pace at which the tasks occurred? Was the pace slow and leisurely or rapid and frantic? |
| Performance | 1-10  Good/Poor | How successful do you think you were in accomplishing the goals of the task set by the experimenter (or yourself)? How satisfied were you with your performance in accomplishing these goals? |
| Effort | 1-10  Low/High | How hard did you have to work (mentally or physically) to accomplish your level of performance? |
| Frustration level | 1-10  Low/High | How insecure, discouraged, irritated, stressed and annoyed versus secure, gratified, content, relaxed and complacent did you feel during the task? |
